# Undetected Severe Fetal Myelosuppression following Administration of High-Dose Cytarabine for Acute Myeloid Leukemia: Is More Frequent Surveillance Necessary?

**DOI:** 10.1155/2017/5175629

**Published:** 2017-09-17

**Authors:** Jessica Parrott, Marium Holland

**Affiliations:** Division of Maternal Fetal Medicine, Department of Obstetrics and Gynecology, University of Kansas School of Medicine, 3901 Rainbow Boulevard, Kansas City, KS 66160, USA

## Abstract

**Background:**

Cytarabine use during pregnancy carries a 5–7% risk of neonatal cytopenia. We report two cases of fetal myelosuppression following high-dose cytarabine administration for acute myeloid leukemia (AML).

**Case 1:**

A 36-year-old G9P6 diagnosed with AML at 21 weeks was monitored for fetal anemia weekly and growth monthly. At 33 weeks (after 2 cycles), BPP was 2/10 and MCA PSV was elevated at 1.51 MoM. Urgent cesarean section was performed. The infant had an initial pH of 6.78 and pancytopenia (hematocrit 13.3%, platelets 3 K/UL, and white blood cell count 2.0 K/UL). Initially transfusion dependent, the neonate had count recovery by 3 weeks.

**Case 2:**

A 30-year-old G4P3 with AML at 26 weeks was monitored for fetal anemia twice weekly and growth monthly. At 34 weeks (after cycle 1), she was admitted with neutropenic fever. The fetal MCA PSV was borderline at 1.48 MoM. It improved to 1.38 MoM at 35 weeks but the fetal tracing worsened. At delivery the fetus was found to have a hematocrit of 30%, but with normal platelet and WBC. The fetus did not require any transfusions.

**Conclusion:**

Cytarabine use during pregnancy may cause neonatal myelosuppression. We recommend monitoring for fetal anemia with MCA Dopplers twice weekly.

## 1. Introduction

Leukemia in pregnancy is found in approximately 1 of every 75,000–100,000 pregnancies with acute myeloid leukemia (AML) accounting for greater than two-thirds of these. An estimated 75% of acute leukemias will be diagnosed in the second or third trimester of pregnancy [1]. Treatment via chemotherapy in the second and third trimester demonstrates increased risks of intrauterine growth restriction (IUGR), intrauterine fetal death (IUFD), neonatal sepsis or death, and neonatal cytopenias (5–7%) [2]. Due to the potential fetal risks of growth restriction and anemia, obstetric management typically includes serial ultrasounds to monitor growth and fetal well-being. Currently however, there are no recommendations as to the frequency with which middle cerebral artery (MCA) Dopplers should be performed to monitor for fetal anemia. We present two cases of AML diagnosed in the second trimester of pregnancy, both of which received HIDAC chemotherapy. The first was complicated by near-fatal fetal pancytopenia and metabolic acidosis necessitating early delivery and the second complicated by isolated fetal anemia.

## 2. Case  1

A 36-year-old gravida 9 para 6 at 21 weeks and 2 days presented with progressive fatigue and malaise. She was found to have anemia (hemoglobin 9.1 gm/dl), thrombocytopenia (platelets 78 k/UL), and 6% circulating blasts on peripheral smear. Bone marrow biopsy confirmed diagnosis of acute myeloid leukemia (FAB classification M2) showing 8% blasts with evidence of dysplasia and hypercellular marrow. FISH was positive for t(8; 21); PML/RaRa and C-KIT were negative. After obtaining a baseline echo, she was started on induction chemotherapy, 7 + 3 (cytarabine 100 mg/m^2^/day continuous infusion for 7 days + daunorubicin 90 mg/m^2^/day intravenous days 1–3). The mother had a biphasic nadir at 7–9 days and at 15–24 days. She was found to be platelet refractory with a positive platelet antibody and required HLA-matched platelets; thus, she remained inpatient until count recovery. Repeat bone marrow biopsy showed both morphologic and multiphase flow cytometry remission. She subsequently was admitted at 27 weeks and 6 days and underwent cycle 1 of consolidation therapy with high-dose cytarabine 3 g/m^2^/day continuous infusion for 6 days. At 31 weeks 6 days, she was admitted for cycle 2 of high-dose cytarabine.

**Figure 1:**
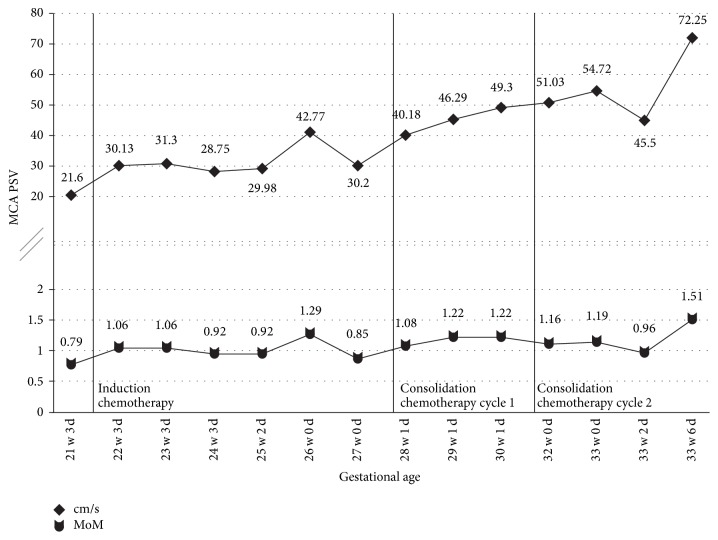
MCA PSV measurements for Case  1. Vertical lines indicate the time of chemotherapy administration. PSV cm/s, peak systolic velocity measurement; MoM, multiples of mean of PSV measurement for corresponding gestational age.

The fetus was monitored twice daily with nonstress tests while being admitted to the hospital for chemotherapy. The pregnancy was followed with serial growth ultrasounds every 4 weeks with weekly Doppler ultrasounds of both the middle cerebral artery (MCA) and umbilical artery.  Figure 1 shows the MCA peak systolic velocity (PSV) trend from 21 weeks until delivery. She was treated with dexamethasone IV twice daily for 3 days for fetal lung maturity at 23 weeks. Her pregnancy was complicated by gestational diabetes diagnosed at 27 weeks that was managed with insulin. She had excellent glycemic control during pregnancy with fasting values less than 85 mg/dL and postprandial values less than 110 mg/dL. At 32 weeks, the daily fetal monitoring had moderate variability but the fetus never demonstrated 15 × 15 reactivity. Thus, BPPs were performed every 2–4 days, which were either 6/10 (−2 breathing, −2 NST) or 8/10 (−2 NST).

At 33 weeks and 6 days (cycle day 15), the fetus appeared to rapidly decompensate over the course of the day. BPP in the morning was 6/10 (−2 breathing, −2 NST). Throughout the day, the fetal heart rate became progressively less reassuring with minimal variability. Repeat BPP in the afternoon was 2/10 (+2 for amniotic fluid index), with centralized MCA peak index (<2.5%) and elevated MCA PSV (1.51 MoM). There was no ultrasound evidence of fetal hydrops or cardiac decompensation. The umbilical artery and ductus venosus Dopplers were normal. Shortly thereafter, the fetal heart rate tracing appeared to become sinusoidal; this resolved, however there was absent to minimal variability with prolonged decelerations. The decision was made to proceed with delivery. Given the extreme maternal pancytopenia with a platelet count of 11 k/UL, a joint decision with haematology was made to delay delivery until after infusion of a loading dose of aminocaproic acid (Amicar, Akorn) and 1 pack of platelets prior to cesarean section. She also received 2 units of packed red blood cells at this time. Cesarean section was performed under general anesthesia 2 hours later with a continuous aminocaproic acid infusion and platelet infusion. Prior to induction of anesthesia the fetal heart rate was 125–130 bpm with absent variability, however initial Apgar was 0 and there was no detectable heart rate. Extensive neonatal resuscitation was performed, requiring intubation, chest compressions, and endotracheal tube epinephrine. Blood gas of the umbilical artery was pH 6.78, with a pCO_2_ tension of 84 mm Hg, pO_2_ tension of 27 mm Hg, and base deficit of 20.9, respectively. There was no evidence of placental abruption at the time of delivery and placental pathology showed focal mild chronic inflammation and chronic infarct without chorioamnionitis or funisitis. Initial labs in the neonatal ICU showed pancytopenia with hematocrit of 13.3%, platelets 3 K/UL and white blood cell count of 2.0 K/UL. On exam, the infant was pale, hypotensive, and with scattered petechiae and bruising. To stabilize the infant, he required transfusions of packed red blood cells, platelets, and fresh frozen plasma for severe pancytopenia; dopamine infusion for hypotension and poor perfusion; surfactant for respiratory distress; and bicarbonate for persistent acidosis.

The infant's pancytopenia slowly resolved over the first 3 weeks, requiring a total of 3 blood transfusions and 7 platelet transfusions. There was no evidence of malformations. Postnatal echocardiogram showed severe septal wall hypertrophy, right ventricle hypertrophy, and hyperdynamic left ventricular function with mid-cavity obliteration at the level of the papillary muscles. Pediatric cardiology was consulted and treated the infant with propranolol. Serial echocardiograms showed slow resolution of cardiac hypertrophy and improvement in left ventricular function with normalization around 1 month of life. Immediate postnatal head ultrasound was negative for intraventricular hemorrhage. Seizure-like activity and concern for encephalopathy was noted initially but EEG was negative. Head MRI however at approximately 2 weeks of life showed numerous microhemorrhagic foci predominantly involving the cerebellum as well as a small subacute subarachnoid hemorrhage. The infant's immediate postnatal course was also complicated by oliguria with acute renal failure and tubular necrosis; max creatinine was 6.17 mg/dL at 2 weeks of life. At 2 months of life, the creatinine had improved to 1.5 mg/dL.

The mother tolerated the surgery well with a total blood loss of 1500 cc. She received aminocaproic acid and oxytocin infusion for 24 hours after surgery with no bleeding complications. Her postoperative course was complicated by a wound infection. The incision was opened 1.5 cm just inferior to the umbilicus and was allowed to heal by secondary intention and oral antibiotic therapy. She has since completed her consolidation chemotherapy and is currently in remission.

## 3. Case  2

**Figure 2:**
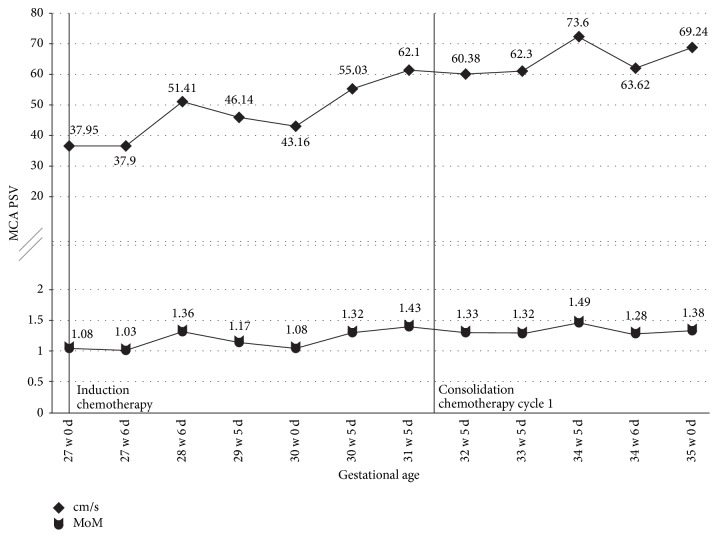
MCA PSV measurements for Case  2. Vertical lines indicate the time of chemotherapy administration. PSV cm/s, peak systolic velocity measurement; MoM, multiples of mean of PSV measurement for corresponding gestational age.

A 30-year-old G4P3003 at 26 weeks pregnant presented to urgent care with nonspecific symptoms and fatigue. Laboratory evaluation revealed pancytopenia. She established prenatal care and repeat labs showed persistent pancytopenia, raising concern for a haematologic malignancy. A subsequent bone marrow biopsy confirmed acute myelogenous leukemia. She was admitted to the hospital and started on induction chemotherapy, 7 + 3 (cytarabine 100 mg/m^2^/day continuous infusion for 7 days + daunorubicin 90 mg/m^2^/day intravenous days 1–3). Maternal fetal medicine was also consulted on admission. She was counseled on the risks and her pregnancy was followed with serial growth ultrasounds and weekly Doppler ultrasounds (see Figure 2) to monitor for fetal growth restriction and fetal anemia. She tolerated the chemotherapy well and subsequently received high-dose cytarabine for consolidation therapy (cytarabine 3 g/m^2^/day continuous infusion for 6 days).

At 34 2/7 weeks, she was admitted for neutropenic fever (cycle 1, day 19). Fetal monitoring was nonreactive but with moderate variability, occasional variable decelerations. The patient's hemoglobin was 5.8 with platelet count of 39 K/UL. She received platelet and blood transfusions with subsequent improvement in fetal tracing. At 34 5/7 weeks, the fetal MCA PSV was noted to have risen to a borderline value, at 1.48 MoM (previously always within normal limits). Fetal growth was normal at the 33rd percentile. At 34 6/7 weeks, the fetal MCA PSV improved (1.28 MoM) but the fetal tracing worsened with decreased variability and increase in the number of variable decelerations. At 35 0/7 weeks, the MCA PI was now centralized with a MCA PSV of 1.38 MoM; a biophysical profile was found to be 6/10, and with continued worsening in the fetal tracing, the decision was made to proceed with delivery.

She underwent an uncomplicated repeat cesarean section with no bleeding complications. Her postoperative course was unremarkable. The infant was born with anemia (hematocrit 30%) but did not require any blood transfusions. The platelet count (274 K/UL) and white blood cell count (7.7 K/UL) were normal at birth. The infant was discharged on hospital day 7. The patient is currently undergoing work-up for a bone marrow transplant.

## 4. Comment

AML in pregnancy is a rare entity, with most of our data coming from case reports. In 2015, the British Journal of Haematology published guidelines for diagnosis and management of AML in pregnancy based on the compiled data available. AML is diagnosed and treated by the same guidelines used in nonpregnant individuals [3]. The recommended treatment consists of induction chemotherapy with cytarabine and an anthracycline (typically daunorubicin) to achieve complete remission, followed by consolidation therapy with high-dose cytarabine or hematopoietic stem cell transplantation [1]. Due to increased maternal mortality, decreased survival time, and reduced remission rates with delay in therapy, the recommendation is to proceed with treatment [1].

We know that both cytarabine and daunorubicin cross the placental barrier; however, with a higher molecular weight and hydrophilic properties, daunorubicin has an incomplete transfer. Cytarabine has low protein binding and wide tissue distribution, including the amniotic cavity [4]. Chemotherapy in the first trimester is associated with an increased risk of congenital malformations and risk of miscarriage (approximately 20%) and it is therefore recommended that the pregnancy be terminated prior to treatment when diagnosis occurs in the first trimester. Administration of cytarabine in the second and third trimesters carries a risk of IUGR (~13%), IUFD (~6%), preterm birth, and neonatal cytopenias [1–3]. It is unknown whether treatment with an anthracycline during pregnancy has potential cardiotoxicity to the fetus as it does in adults. Limited studies using daunorubicin with long-term follow-up of these children have shown no cardiac damage [1]. Leukemia itself also has an impact on perinatal outcome, despite treatment. There are known associations of spontaneous abortion, prematurity, IUGR, and IUFD [2]. This increased risk of fetal death is not completely understood. Postulated etiologies include maternal anemia, disseminated intravascular coagulation and leukemic cells affecting blood flow, and nutrient and/or oxygen exchange in the intervillous spaces of the placenta [3].

Here we present one case complicated by severe neonatal pancytopenia and a second complicated by moderate neonatal anemia. Two studies examining the maternal and neonatal outcomes of pregnant women with AML [5] or those receiving chemotherapy treatments [2] report a 5–7% risk of neonatal cytopenias. All of these cases received cytarabine either alone or in combination with 1 or more chemotherapeutic agents [2, 5]. With immature liver and kidneys, neonates have a limited capacity to metabolize and eliminate drugs which is part of the reason we do not recommend administering chemotherapy after 35 weeks [1]. So although each case describes a transient myelosuppression, it may last 3-4 weeks which can place the neonate at substantial risk for complications such as sepsis [2, 5–8]. Thus, while the overall risk of neonatal cytopenias is low, it can have a profound effect leading to multiple transfusions and potential complications.

Utilizing MCA peak systolic velocity (PSV), fetal anemia can be diagnosed via noninvasive methods. Studies have demonstrated that a threshold of 1.5 MoM is suggestive of moderate to severe fetal anemia [9]. In 2014, a study evaluated the use of MCA Dopplers for monitoring of fetal anemia when chemotherapy is administered to pregnant women with ovarian, cervical, or breast cancer. They measured the MCA PSV one day before and the third day after chemotherapy. No severe range fetal anemia was diagnosed but they found a number of cases of mild fetal anemia which supports the use of MCA Dopplers in monitoring pregnancies treated with chemotherapy [9]. The 2015 guidelines published in the British Journal of Haematology recommend serial ultrasounds every 4 weeks for growth and fetal well-being to monitor for growth restriction. They do not, however, comment on monitoring for fetal anemia [3]. In contrast, a 2014 review article published in the European Journal of Haematology recommends checking for fetal anemia before and after chemotherapy but does not provide any guidance as to when this should be performed [1]. Clearly, the data supports monitoring for fetal anemia but guidance is needed regarding timing and frequency.

For our first case, we decided to screen for fetal anemia weekly with MCA Dopplers on ultrasound. Despite this frequency of monitoring, it was the fetus developing a metabolic acidosis as demonstrated by the nonreassuring fetal heart rate tracing that prompted us to repeat the MCA Dopplers and discover the fetal anemia. If we had been monitoring at an increased frequency, such as twice weekly, we might have noticed an increasing trend or caught the anemia at an earlier date and been able to intervene at that time, possibly preventing the neonatal hypoxic event and severe pancytopenia seen at birth. With our second case, we chose to monitor the MCA Dopplers twice weekly. At the time of delivery mild to moderate neonatal anemia was present but did not require any interventions.

In both cases, there was never any ultrasound evidence of hydrops fetalis or cardiac decompensation. The umbilical artery and ductus venosus Doppler studies were normal throughout the entire pregnancy for both women. The Doppler interrogations were all performed with the same methodology; however, the person performing each ultrasound was not always the same. We do not believe that this would have contributed to any difference in findings. It is not unreasonable to suspect that the more significant fetal compromise seen in the first case could be related to receiving 2 cycles of consolidation therapy with the high-dose cytarabine compared to the 1 cycle received in the second case; however, there is no current literature to support this theory.

While the risk of neonatal cytopenia is low, the impact can be significant as evidenced by this case. We have a noninvasive method for screening for fetal anemia that should be utilized with pregnant women receiving chemotherapy. With medications that induce a prolonged period of maternal myelosuppression, such as cytarabine, we would recommend monitoring the pregnancy with serial ultrasounds and MCA Dopplers at least twice weekly during the period of maternal myelosuppression. If there is concern for progression of fetal anemia and development of hydrops fetalis, the frequency of monitoring should be increased and delivery may need to be considered.
